# Occlusal force is correlated with cognitive function directly as well as indirectly via food intake in community-dwelling older Japanese: From the SONIC study

**DOI:** 10.1371/journal.pone.0190741

**Published:** 2018-01-05

**Authors:** Kazunori Ikebe, Yasuyuki Gondo, Kei Kamide, Yukie Masui, Taturo Ishizaki, Yasumichi Arai, Hiroki Inagaki, Takeshi Nakagawa, Mai Kabayama, Hirochika Ryuno, Hitomi Okubo, Hajime Takeshita, Chisato Inomata, Yuko Kurushima, Yusuke Mihara, Kohdai Hatta, Motoyoshi Fukutake, Kaori Enoki, Taiji Ogawa, Ken-ichi Matsuda, Ken Sugimoto, Ryosuke Oguro, Yoichi Takami, Norihisa Itoh, Yasushi Takeya, Koichi Yamamoto, Hiromi Rakugi, Shinya Murakami, Masahiro Kitamura, Yoshinobu Maeda

**Affiliations:** 1 Department of Prosthodontics, Gerodontology and Oral Rehabilitation, Osaka University Graduate School of Dentistry, Osaka, Japan; 2 Department of Clinical Thanatology and Geriatric Behavioral Science, Osaka University Graduate School of Human Sciences, Osaka, Japan; 3 School of Allied Health Sciences, Osaka University Graduate School of Medicine, Osaka, Japan; 4 Tokyo Metropolitan Institute of Gerontology, Research Team for Human Care, Tokyo, Japan; 5 Keio University, School of Medicine, Tokyo, Japan; 6 University of Zurich, University Research Priority Program "Dynamics of Healthy Aging", Zurich, Switzerland; 7 Department of Health Promotion, National Institute of Public Health, Saitama, Japan; 8 Department of Geriatric and General Medicine, Osaka University Graduate School of Medicine, Osaka, Japan; 9 Department of Periodontics, Osaka University Graduate School of Dentistry, Osaka, Japan; UNIVERSITY OF GRANADA, SPAIN

## Abstract

**Background:**

Growing evidence suggests that oral health may be an important factor associated with cognitive function in aged populations. However, many previous studies on this topic used insensitive oral indicators or did not include certain essential covariates. Thus, we examined the association between occlusal force and cognitive function in a large sample of older adults, controlling for dietary intake, vascular risk factors, inflammatory biomarkers, depression, and genetic factors.

**Methods:**

In this cross-sectional study of older community-dwelling Japanese adults, we examined data collected from 994 persons aged 70 years and 968 persons aged 80 years. Cognitive function was measured using the Japanese version of the Montreal Cognitive Assessment (MoCA-J). Oral status and function were evaluated according to the number of remaining teeth, periodontal pocket depth, and maximal occlusal force. Associations between MoCA-J scores and occlusal force were investigated via bivariate and multivariate analyses.

**Results:**

Education level, financial status, depression score, and intake of green and yellow vegetables, as well as number of teeth and occlusal force, were significantly correlated with MoCA-J scores in both age groups. Among individuals aged 80 years, CRP and periodontal status were weakly but significantly associated with MoCA-J score. After controlling for all significant variables via bivariate analyses, the correlation between maximal occlusal force and cognitive function persisted. A path analysis confirmed the hypothesis that cognitive function is associated with occlusal force directly as well as indirectly via food intake.

**Conclusions:**

After controlling for possible factors, maximal occlusal force was positively associated with cognitive function directly as well as indirectly through dietary intake.

## Introduction

Cognitive impairment is a substantial health concern for rapidly aging societies, where it is a major cause of severe disability. In the last year of life, 68% of patients with advanced dementia were labeled as having a persistently severe disability, a value that far exceeds the percentage of those with frailty (25%) or organ failure (18%) [1].

The number of people with dementia is expected to nearly double in the next 20 years over the world [2]. A Japanese survey conducted in 2012 indicated that 15% of the older population (65 year and older) had been diagnosed with dementia, while 13% had mild cognitive impairment (MCI). Approximately half of individuals with MCI progress to dementia within 5 years of the initial diagnosis. Therefore, strategies for preventing or delaying the onset of dementia, even marginally, are extremely important.

In an evaluation of relative risks from existing meta-analyses, Norton et al. reported the following potentially modifiable risk factors: 1) fewer years of education, which carried the greatest risk, 2) vascular risk factors (for example, smoking, physical inactivity, hypertension, diabetes, and obesity), and 3) depression [3]. These risk factors were mutually related, resulting in a complex etiological model. Growing evidence suggests that dental health may be an important factor associated with cognitive function in older populations. In a co-twin control analysis, researchers found that, after excluding genetic and common environmental risk factors, tooth loss before age 35 was the only significant risk factor for Alzheimer's disease [4]. This suggests that tooth loss from early adulthood and midlife onwards contributes to the risk of dementia.

Several models have been proposed to describe the mechanisms underlying the relationship between cognitive decline and oral health [5, 6]. The association between oral health and cognitive function can be confounded by several common risk factors, such as age, lifestyle, and systemic diseases. Optimally, all possible factors should be included in a comprehensive model; however, no studies have evaluated such a wide range of variables in one group of individuals via concurrent examination by experts in each field. Cerutti-Kopplin et al. [7] published a review article in which the authors summarized three plausible mechanisms that may explain the association between oral health and cognitive impairment: 1) periodontal disease as systemic inflammation 2), reduced mastication-induced sensory stimulation to the brain, and 3) poor nutritional intake. However, they pointed out that previous studies did not contain comprehensive nutritional evaluations or data on inflammatory biomarkers.

Previously, studies investigating the relationship between cognitive function and oral health have mainly focused on periodontal disease [8], and not impaired mastication. However, in the last decade, researchers have increasingly focused on mastication in the context of cognitive impairment [5, 6]. Unfortunately, few of these studies have objectively measured masticatory function. As deteriorated masticatory function can affect nutritional intake, the relationship among mastication, nutrition, and cognitive impairment should be clarified. However, no studies on mastication and cognitive function have included all of the relevant variables with respect to inflammatory, dietary, and masticatory conditions.

Therefore, we sought to examine the association between occlusal force in full dental arches, including artificial teeth, and cognitive function in a large sample of functionally independent older Japanese adults. We also examined periodontal condition and dietary intake, adjusted for vascular risk factors, inflammatory biomarkers, depression, and a genetic factor. We hypothesized that, after adjusting for possible risk factors, occlusal force would be significantly associated with cognitive function, mediated by dietary intake, among functionally independent older Japanese adults.

## Methods

The study protocol and informed consent documents were approved by the Institutional Review Board (IRB) at the Osaka University Graduate School of Dentistry (approval number H22-E9). Written informed consent was obtained from all the participants.

### Study population and procedure

This study encompassed a cross-sectional examination of data collected during the baseline assessment component of a prospective study of health and longevity called ‘Septuagenarians, Octogenarians, and Nonagenarians Investigation with Centenarians’ (SONIC) study [9, 10]. The participants were older adults living in private residences. Their ages ranged from 69–71 years (classified into a ‘70 years group’, n = 994) or 79–81 years (‘80 years group’, n = 968). The initial purpose of the narrow-age cohort design was to investigate individual differences in each age group. The recruiting procedure is detailed elsewhere [10].

The participants were invited to come to the examination venue in groups of 30 to 50 participants per day. All examinations for each participant were completed in 1 day. Dentists (periodontists and prosthodontists) examined dental status and oral function. A registered dietitian assessed dietary intake. A psychologist examined socioeconomic status, psychological status, cognitive function, and depression status. Physicians and nurses obtained a history of chronic conditions, measured blood pressure, and collected blood samples.

Participants with no occlusal contact between their own teeth or prostheses were excluded from the study due to the impossibility of measuring their occlusal force. Additionally, participants with dementia who could not complete the MoCA-J test were excluded.

### Number of remaining teeth, periodontal pocket depth

Registered dentists performed all dental examinations with a dental mirror and a dental explorer. The periodontal pocket depth (PPD) of each tooth was measured using a dental CP-12 (color-coded probe, Hu-Friedy Mfg. Co LLC, Chicago). PPD was assessed at six sites (mesio-buccal, mid-buccal, disto-buccal, mesio-lingual, mid-lingual, and disto-lingual) for all teeth present. We evaluated the severity and spread of the periodontal disease based on the maximal PPD (mm), mean PPD (mm), and percentage of teeth with PPD ≥ 4 mm. Unfortunately, we were unable to conduct X-ray analyses during this field examination, and information regarding alveolar bone resorption was not obtained. Thus, it was difficult to strictly diagnose gingivitis or periodontitis. Taking this into consideration, we avoided using the term “periodontitis” and “periodontal status” in this manuscript. PPD was assessed at six sites for all teeth present. Mean PPD and maximum PPD were used as indicators of periodontal status [11].

### Maximal occlusal force

Masticatory function can be assessed via chewing tests and questionnaires. Whereas chewing tests enable the assessment of masticatory efficiency and performance with some degree of objectivity, questionnaires help with the evaluation of individual subjective responses regarding chewing ability [12]. Masticatory efficiency pertains to the number of masticatory cycles required to reduce foods to a certain size. Masticatory performance, which is the most common and powerful measure used, pertains to the particle size distribution of food chewed in a standard number of cycles [13].

We used maximal occlusal force as a proxy measurement because it is strongly correlated with objectively measured masticatory performance [14–16] and can be tested in a number of seconds. The bilateral maximal occlusal force between full dental arches was measured using 97-μm-thick pressure-sensitive sheets (Dental Prescale 50H R type; Fuji Film Co., Tokyo, Japan). The participants performed maximal clenching in the intercuspal position for 3 seconds with the pressure-sensitive film placed between the maxillary and mandibular dental arches. Participants with removable partial dentures kept their dentures in place during the measurement. The inter-examiner and intra-examiner reliability of this type of measurement has been previously described [10].

### Cognitive function

We used the Japanese version of the Montreal Cognitive Assessment (MoCA-J) [17] as a general index of cognitive status [18]. The MoCA is a brief cognitive screening tool developed originally for detecting mild cognitive impairment (MCI) in older people [19]. The MoCA-J has demonstrated good reliability and validity for predicting early cognitive decline compared with conventional cognitive tests [17]. Thus, we used the MoCA-J total score (0 to 30 points) as a measure of cognitive function. A higher MoCA-J score reflects higher cognitive function.

### Dietary assessment

Dietary habits during the preceding month were assessed using a validated, brief-type self-administered diet history questionnaire (the BDHQ) [20, 21]. The BDHQ is a structured, fixed-portion questionnaire that collects information about the consumption frequency of selected foods commonly consumed in Japan, general dietary behavior, and usual cooking methods. Because most of the participants were physically and cognitively healthy, they reported preparing meals in their homes. The majority of participants were able to physically fill out the questionnaire without assistance.

Daily food (58 food and beverage items in total), energy, and selected nutrient intake were calculated using an ad hoc computer algorithm for BDHQ, which was based on the Standard Tables of Food Composition in Japan [22]. Detailed descriptions of the methods used to calculate dietary intake and the validity of the BDHQ have been published previously [20, 21]. The value of food intake was energy-adjusted using the density method (that is, amount per 1000 kcal of energy) to minimize the influence of dietary misreporting. In this study, we used food intake (g/1000 kcal) of the following different foods as dietary variables: grains, potatoes, pulses, green and yellow vegetables, other vegetables, fruits, fish and shellfish, meats, eggs, and dairy products.

### Chronic conditions

Hypertension diagnoses were based on blood pressure values greater than 140/90 mmHg and/or whether the individual was receiving antihypertensive treatment [23]. Diabetes was defined by fasting plasma glucose concentrations ≥ 7.0 mmol/L (126 mg/dL), casual plasma glucose concentrations ≥ 11.1 mmol/L (200 mg/dL), HbA1c ≥ 6.5%, or current pharmaceutical treatment for diabetes, according to the World Health Organization criteria for epidemiologic studies of diabetes. CRP > 0.3 mg/dL is a stratification factor related to systemic inflammation.

### ApoE genotype assessment

ApoE-ε4 carriers have a higher risk of developing the disease, and tend to be afflicted with early onset Alzheimer’s [24]. Therefore, we analyzed data regarding Apolipoprotein E (ApoE) genotyping using a blood sample from a vein. Individuals with at least one ε4 allele were labeled ApoE-ε4 positive.

### Other recorded variables

Participants were interviewed to collect information about education level (junior high school; high school; college or higher), self-rated financial status (good; fair; poor), current drinking habits (yes; no), smoking history (yes; no), and body mass index (BMI). Participants with a BMI of 25 or over were classified as overweight. Depression was evaluated using the 5-item short form of the Geriatric Depression Scale (GDS-5: score range 0–5) [25].

### Statistical analysis

Due to the numerous measurements in various scientific fields, and statistical methods used, a large sample size was necessary to produce adequate statistical power. In order to detect a minimally meaningful effect size, i.e., f2 = 0.02 for coefficient of determination in a multiple linear regression model, the sample size was required to be about 878 by G*power [26] when α error was 0.05, Power = 1-β error was 0.80, and the number of predictors including covariates was 12. Therefore, the number of study participants in both the 70 and 80years groups was sufficient for our study.

First, we conducted univariate analyses with t-tests, ANOVAs, and bivariate analyses with Spearman’s rank-order correlation coefficient to evaluate the association between cognitive function and other potentially-related variables in the 70 and 80 years groups.

For our analysis of dietary intake, we excluded participants with extremely low or high reported energy intake (< 600 or ≥ 4000 kcal/d), those currently receiving dietary counseling from a doctor or dietician, and those who had undergone an intentional dietary change during the preceding year. As some participants were in more than one exclusion category, the final analysis sample comprised 805 participants in the 70 years group and 793 participants in the 80 years group.

Second, we performed multiple linear regression analyses of the total sample. The outcome variable was MoCA-J score, the explanatory variable was maximal occlusal force, and the controlled variables were number of teeth, periodontal status, socioeconomic status, lifestyle, chronic conditions, depression, inflammatory condition (CRP), and dietary intake. *P* < 0.05 was considered to denote a statistically significant difference. The models including the number of teeth and periodontal status were initially adjusted for age and gender (Model 1). In Model 1, the number of teeth and periodontal status were not significantly associated with MoCA-J score. Thus, only the occlusal force was included in the next model, which was also adjusted for socioeconomic status (Model 2). Edentulous participants (70 years group, n = 51; 80 years group, n = 143) were excluded in Model 1 but included from Model 2 to 4. We then added adjustments for confounding variables found to be significant in the bivariate analyses (Model 3), and finally adjusted for dietary intake (Model 4).

All analyses were performed using SPSS statistical software version 21 (SPSS Inc., Chicago, IL, USA), with a significance level of 5%.

Finally, to examine the indirect effect of dietary intake on the relationship between occlusal force and cognitive function, and to examine the hypothesized model fit, we conducted path analysis. Path analysis can be used instead of several separate regressions to examine mediating effects within a single model [27]. Additionally, path analysis allows the testing of causal relationships among a set of observed variables. The analysis was undertaken using AMOS 21 software (IBM). Chi-square statistics, root mean square error of approximation (RMSEA), and the goodness of fit index (GFI) were used as fit indices.

## Results

The MoCA-J scores for participants in the 70 years group was significantly higher than those in the 80 years group. Table 1 shows a comparison of MoCA-J scores according to socioeconomic status, lifestyle, medical history, obesity, CRP, and presence/absence of the ApoE-ε4 allele. Among the 70 years group, male participants with a lower education level, lower financial status, a smoking history, hypertension, diabetes, and overweight status had significantly lower MoCA-J scores. Among the 80 years group, only the participants with a lower education level, lower financial status, and higher CRP had significantly lower MoCA-J scores. However, we found no significant differences in MoCA-J scores related to the other variables, including ApoE genotype.

**Table 1:** Comparison of MoCA-J score by demographic/health variables.

	70 years group (n = 994)						80 years group (n = 968)					
	% of study population	MoCA-J score				p- value	% of study population	MoCA-J score				p- value
		mean	95% CI					mean	95% CI			
Variables	100.0	22.7	22.5	-	22.9		100.0	21.1	20.8	-	21.3	
Gender ^#^												
Male	47.9	22.4	22.1	-	22.7	0.006	47.0	21.3	20.9	-	21.6	0.164
Female	52.1	23.0	22.7	-	23.3		53.0	20.9	20.6	-	21.2	
Education level^##^												
Junior high school	29.2	20.9	20.5	-	21.3	<0.001	32.0	19.4	18.9	-	19.8	<0.001
High school	43.1	23.2	22.9	-	23.5		39.9	21.5	21.1	-	21.9	
College or more	27.8	23.9	23.6	-	24.3		28.1	22.4	22.0	-	22.8	
Self-rated financial status^##^												
Poor	22.8	21.9	21.5	-	22.4	<0.001	19.3	20.2	19.6	-	20.7	<0.001
Fairly good	53.5	22.7	22.4	-	23.0		57.1	21.0	20.7	-	21.3	
Good	23.7	23.4	23.0	-	23.8		23.7	22.0	21.5	-	22.5	
Smoking history^#^												
Yes	39.7	22.3	22.0	-	22.7	0.003	39.2	21.1	20.7	-	21.5	0.994
No	60.3	23.0	22.7	-	23.3		60.8	21.1	20.7	-	21.4	
Drinking habit^#^												
Yes	32.2	22.8	22.4	-	23.2	0.912	29.4	21.2	20.7	-	21.6	0.583
No	67.8	22.8	22.5	-	23.0		70.6	21.0	20.7	-	21.3	
Hypertension^#^												
Yes	66.4	22.5	22.3	-	22.8	0.003	81.9	21.1	20.8	-	21.4	0.734
No	33.6	23.2	22.9	-	23.6		18.1	21.0	20.4	-	21.6	
Diabetes^#^												
Yes	18.5	22.1	21.6	-	22.7	0.023	16.5	20.9	20.3	-	21.5	0.627
No	81.5	22.8	22.5	-	23.0		83.5	21.1	20.8	-	21.4	
Overweight^#^												
Yes	21.1	22.0	21.4	-	22.5	<0.001	19.5	21.3	20.8	-	21.9	0.256
No	78.9	22.9	22.7	-	23.1		80.5	21.0	20.7	-	21.3	
CRP^#^												
≦0.3 mg/dL	91.8	22.7	22.5	-	23.0	0.070	89.6	21.2	20.1	-	21.5	0.001
0.3 mg/dL<	8.2	22.0	21.3	-	22.8		10.4	19.9	19.1	-	20.7	
ApoE-ε4 allele ^#^												
absence	85.4	22.6	22.3	-	22.8	0.767	87.8	21.2	21.0	-	21.5	0.096
presence	14.6	22.7	22.2	-	23.2		12.2	20.6	19.8	-	21.4	

^#^t-test

^##^ANOVA

CI: confidence interval. Participants with missing data were excluded from analysis.

The correlations between MoCA-J scores and dental status, depression score, and food intake are shown in Table 2. Number of teeth, maximal occlusal force, and depression score were significantly correlated with MoCA-J scores for both the 70 and 80 years groups. Of all the variables associated with periodontal status, the percentage of PPD ≥ 4 mm teeth was the only trait that was significantly correlated with MoCA-J score (weak negative correlation) in the 80 years group. Additionally, none of the three periodontal variables were significantly correlated with CRP in either age group. With respect to food intake, green and yellow vegetables, fruits, and meats were positively correlated with MoCA-J scores while grains were negatively correlated with MoCA-J scores in both age groups.

**Table 2:** Correlations between MoCA-J score and dental status/food intake.

	70 years group (n = 994)			80 years group (n = 968)		
Variables	mean	rs	p-value	mean	rs	p-value
Number of teeth	20.4	0.164	<0.001	15.4	0.080	0.014
Occlusal force (N)	529	0.198	<0.001	326	0.157	<0.001
Maximal PPD^§^ (mm)	5.05	-0.020	0.549	5.15	-0.007	0.841
Mean PPD^§^ (mm)	3.20	-0.022	0.492	3.45	-0.035	0.329
Percentage of PPD ≥4mm teeth^§^ (%)	24.2	-0.044	0.190	36.4	-0.075	0.036
Depression	0.98	-0.096	0.003	1.40	-0.168	<0.001
Food intake (g/1000 kcal)	70 years group^§§^ (n = 805)			80 years group^§§^ (n = 793)		
Grains	211.6	-0.161	<0.001	210.7	-0.082	0.021
Potatoes	31.0	0.097	0.006	34.6	0.034	0.342
Pulses	39.6	0.140	<0.001	38.1	0.047	0.187
Green and yellow vegetables	67.9	0.249	<0.001	69.6	0.101	0.005
Other vegetables	106.6	0.153	<0.001	101.6	0.022	0.546
Fruits	86.1	0.084	0.017	89.6	0.108	0.002
Fish and shellfish	56.8	-0.005	0.887	56.2	0.037	0.297
Meats	31.6	0.108	0.002	30.5	0.090	0.011
Eggs	19.7	0.092	0.009	20.0	0.056	0.118
Dairy products	76.3	0.066	0.062	82.0	0.056	0.118

rs: Spearman rank-order correlation coefficient for MoCA-J score; PPD: periodontal pocket depth. Depression: GDS-5 score (continuous variable)

§: Edentulous participants (70 years group, n = 51; 80 years group, n = 143) were excluded from these correlations.

§§: Participants currently receiving dietary counseling from a doctor or dietician, and those with intentional dietary changes during the preceding year were excluded from analysis.

Table 3 shows the results of multiple linear regression analyses for associations with MoCA-J score (the dependent variable) for both age groups combined. Model 1 indicated that occlusal force was significantly associated with MoCA-J score (Beta: standardized partial regression coefficient = 0.174, p < 0.001); however, the number of teeth and periodontal status were not significantly associated with MoCA-J score. Model 2 showed that after controlling for age group, gender, education level, and self-rated financial status, the occlusal force was still significantly correlated with MoCA-J score (Beta = 0.137, p < 0.001). Model 3 showed that after controlling for Model 2 plus lifestyle and medical variables, the occlusal force was still significantly correlated with MoCA-J score (Beta = 0.139, p < 0.001). Model 4, which included food intake, revealed that ingestion of green and yellow vegetables was significantly and positively related to MoCA-J score. After controlling for all of these variables, the significant association between the occlusal force and MoCA-J score remained (Beta = 0.125, p < 0.001).

**Table 3:** Linear regression models for occlusal force and MoCA-J score, adjusted for significant independent variables.

Occlusal force	Model 1^§^	n = 1681	Model 2	n = 1899	Model 3	n = 1626	Model 4^§§^	n = 1326
	Beta	p-value	Beta	p-value	Beta	p-value	Beta	p-value
Age	-0.163	<0.001	-0.176	<0.001	-0.149	<0.001	-0.163	<0.001
Gender	0.041	0.090	0.051	0.016	0.054	0.092	0.035	0.324
Number of teeth	-0.007	0.814						
Percentage of PPD ≥4mm teeth (%)^§^	-0.036	0.167						
Occlusal force	0.174	<0.001	0.137	<0.001	0.139	<0.001	0.125	<0.001
Education level								
High school			0.256	<0.001	0.245	<0.001	0.242	<0.001
College or more			0.332	<0.001	0.316	<0.001	0.304	<0.001
Financial status								
Fairly good			0.070	0.009	0.076	0.009	0.105	0.001
Good			0.113	<0.001	0.106	<0.001	0. 129	<0.001
Smoking history					0.015	0.608	0.024	0.459
Drinking habit					0.012	0.645	0.021	0.455
Overweight					-0.034	0.135	-0.032	0.198
Hypertension					-0.037	0.110	-0.029	0.244
Diabetes					-0.033	0.148	-0.030	0.226
CRP>0.3 mg/dL					-0.063	0.006	-0.089	<0.001
Depression					-0.079	0.001	-0.082	0.002
Green and yellow vegetables							0.086	0.002
Fruits							-0.011	0.699
Meat							0.037	0.160
Grains							-0.016	0.538
adjusted R^2^	0.078		0.175		0.185		0.212	

Beta: standardized partial regression coefficient. Model 1: Occlusal force, number of remaining teeth, and percentage of teeth with a periodontal pocket depth of 4 mm or more. Model 2: Occlusal force adjusted for gender (male*, female), education level (junior high school*, high school, college or more), self-rated financial status (poor*, fairly good, good). Model 3: Model 2 plus adjustments for smoking history and drinking habits (yes, no*), hypertension (yes, no*), diabetes mellitus (yes, no*), overweight status (BMI: equal to or greater than, less* than 25 kg/m^2^), CRP (equal to or greater than, less* than 0.3 mg/dl) and Depression (GDS-5 score, continuous variable). Model 4: Model 3 plus adjustments for food intake (continuous variable).

*: Reference category

§: Edentulous participants were excluded in Model 1 but included from Model 2 to 4.

§§: Participants currently receiving dietary counseling from a doctor or dietician and those with intentional dietary change during the preceding year were excluded from analysis.

**Fig 1:**
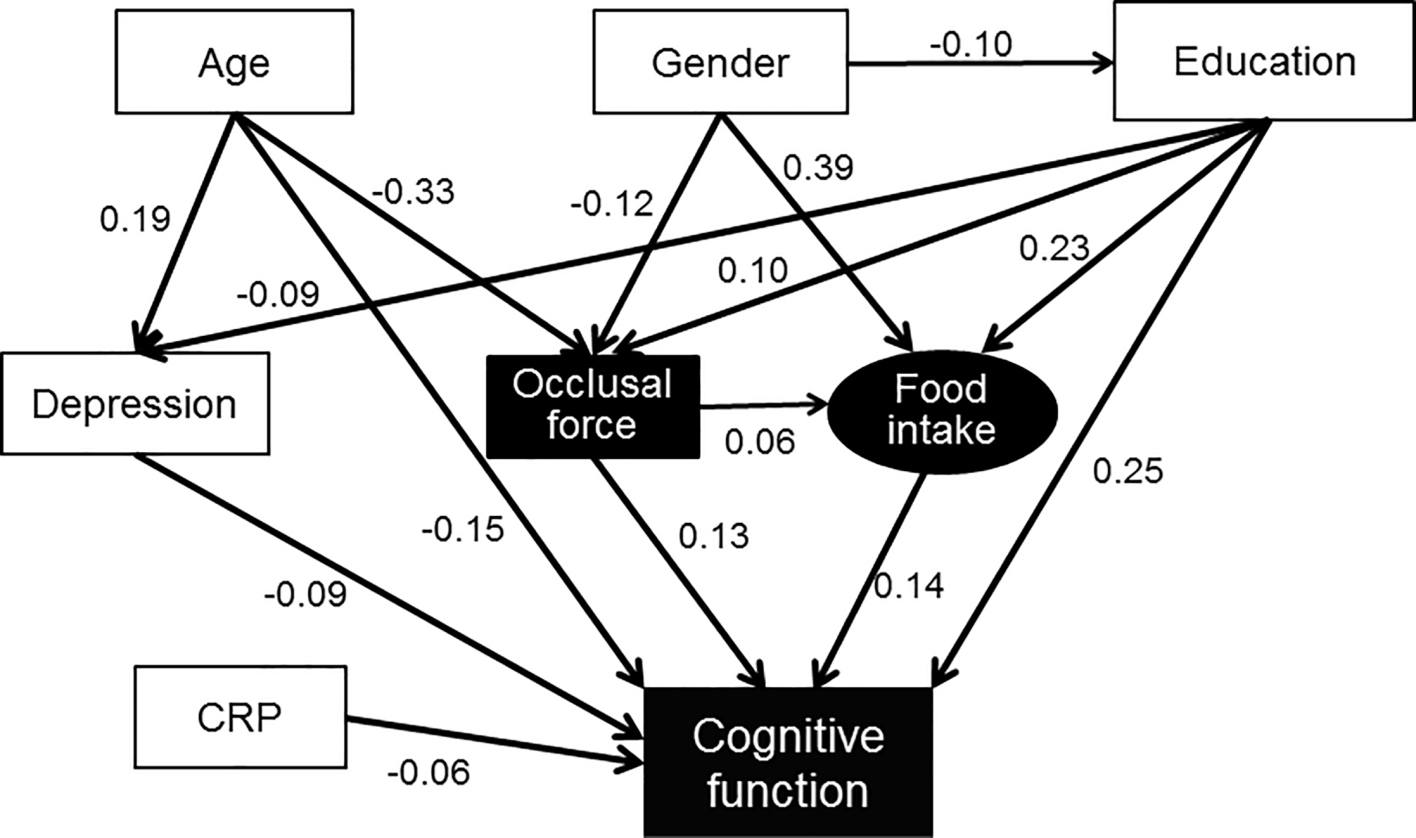
Path analysis from occlusal force to cognitive function. Arrows show significant paths and numbers are significant standardized coefficients (p<0.05). The hypothesized model showed an acceptable good fit (GFI = 0.976, RMSEA = 0.057, CFI = 0.879, χ^2^ = 252.3).

The path analysis, shown in Fig 1, confirmed the hypothesis that cognitive function is associated with occlusal force. The arrows show significant paths and the numbers are standardized coefficients. The path model confirmed that cognitive function is directly associated with educational level, age, depression, micro-inflammation, food intake, and occlusal force. Occlusal force was associated with cognitive function directly as well as indirectly via dietary intake. The path from occlusal force to food intake was weak but statistically significant. The hypothesized model showed an acceptable good fit (GFI = 0.976, RMSEA = 0.057, CFI = 0.879).

## Discussion

To the best of our knowledge, this study is the first investigation of the association between masticatory function and cognitive function among a large sample of older adults. We examined socioeconomic status, lifestyle, vascular risk factors, inflammatory biomarkers, mental status, dietary intakes, and a genetic factor. Although several studies have reported an association between oral health and cognitive function [6, 7, 25], this relationship can be confounded and mediated by several factors that are mutually related, resulting in a complex etiological model. We sought to move towards a comprehensive model of the possible mechanisms underlying this association by including all of the above-mentioned risk factors. Our path analysis estimated that oral function is associated with cognitive function directly as well as indirectly through dietary intake.

In a recent systematic review regarding the association between oral health and cognitive status, Wu et al. concluded that the variable linking oral health with cognitive status is unclear. They stated that methodological limitations play a major role in explaining inconsistent findings, thus, additional research is needed with better investigative strategies [28]. In the present study, we intended to investigate the effect of oral health on cognitive function among functionally independent elderly people. Elderly people are more likely to lose teeth, and subsequently, to receive prosthodontic treatment (e.g. removable dentures, bridges, and dental implants). Thus, it is not possible to evaluate masticatory function solely using information regarding the number of remaining teeth. Thus, we conducted an objective measurement of oral function. It is difficult to measure masticatory performance among a large number of people because of the large time requirement. Therefore, we used the maximal occlusal force between the upper and lower dental arches, including artificial teeth, as a surrogate measure of masticatory function. We believe this measurement is appropriate because maximal occlusal force is highly correlated with objectively measured masticatory performance and takes only a number of seconds to measure [14, 16]. To assess cognitive function in functionally independent older people, we used the Japanese version of the Montreal Cognitive Assessment (MoCA-J). This test has been used to measure global cognitive status in a number of population-based studies [17, 29]. The MoCA may be sensitive to early cognitive changes in multiple domains, as is used to screen mild cognitive impairment in persons performing in the normal range on the MMSE.

Because cognitive function is multi-factorial, data from various fields are useful when investigating the contributing factors. Although many earlier studies on cognitive decline adjusted for socioeconomic status and chronic diseases, they often failed to control for other possible risk factors such as a systemic inflammatory condition, mental health, dietary intake, and genetic factors. As anticipated, our bivariate analyses showed that cognitive function was significantly associated with gender, financial status, education level, lifestyle, depressed state, inflammatory condition, and food intake. Therefore, these variables should be included as confounding factors in models of cognitive function. It is likely that the ApoE genotype was not significantly associated with cognitive function because all of the participants were functionally independent older people, and thus had mild as opposed to more severe cognitive decline.

In our bivariate analyses, number of teeth, periodontal status, and occlusal force were significantly associated with cognitive function. Nevertheless, the number of teeth and periodontal pocket depths became statistically insignificant after adjusting for occlusal force. This implies that the associations between these variables and cognitive function may be confounded by oral function. One previous study showed that, in a regression analysis, multiple tooth loss became insignificant with respect to cognitive impairment while chewing difficulty remained significant. However, tooth loss and chewing ability data in the above study were self-reported and dichotomized [30]. Our methods were taking advantage of using sophisticated examinations for both oral function and cognitive ability.

Several previous articles have reported that periodontal disease or tooth loss (as a possible end point of periodontal disease) is associated with cognitive decline [6, 31]. It has been hypothesized that periodontal disease-derived inflammatory molecules, bacteria, and bacterial products can enhance brain inflammation [32]. Our results showed that, while CRP was weakly but significantly associated with cognitive function, periodontal status was not significantly associated with CRP. Thus, oral function may have a more significant impact on cognitive impairment rather than periodontal inflammation in our participant group. However, simple measurement and classification of periodontal status did not reveal a clear association between periodontal disease and cognitive function or CRP.

We have summarized our findings in the following points. First, our results from the multiple linear regression analyses indicate that occlusal force and intake of green and yellow vegetables were independently related to cognitive function. Second, the model from our path analysis clarified that cognitive function among our participants was mainly associated with age, education level, food intake, and oral function, although increased CRP and depression levels were also significant. Finally, the association between cognitive function and occlusal force decreased when nutritional intake was controlled for among the 70 years group. These findings implied that lower occlusal force (regardless of whether the individual had natural or artificial teeth) might be directly linked to cognitive function, while also affecting dietary intake, and thus be indirectly related to cognitive function.

Risk factors during early adulthood and midlife, such as masticatory deterioration, may contribute to cognitive decline with a cumulative detrimental effect on the brain and nutrient intake. For example, human studies have shown that natural teeth and artificial teeth, as well as jaw movement, give sensory input and motor feedback to the central nervous system [33, 34]. Similarly, the increase in cerebral blood flow, activation of cortical areas, and blood oxygen levels resulting from masticatory stimulation are thought to activate brain function [35, 36]. Several pieces of evidence demonstrate that lack of antioxidant nutrients (vitamins E and C, carotenoids, flavonoids, enzymatic cofactors), homocysteine-related vitamins (B-vitamins), and n-3 polyunsaturated fatty acids (EPA and DHA) can be risk factors for decreased cognitive function [37]. Correspondingly, we found that occlusal force was positively associated with intake of all the antioxidant nutrients, B vitamins, and n-3 polyunsaturated fatty acids in our study participants. Additionally, MoCA-J scores were also significantly correlated with intake of the above-listed nutrients, except for Vitamin A, B_12_, and n-3 polyunsaturated fatty acids. Thus, although the associations in our study are weak, they are detectable and have biological plausibility.

Although older people tend to have fewer teeth and lower occlusal force, we expect that the dual strategy of 1) preventing ongoing tooth loss and 2) providing adequate prosthodontic treatment will help to maintain occlusal force, supplemented by ongoing mental stimulation and optimal dietary intake.

### Limitations

Several aspects of our study design limit our conclusions. The first point of concern is the narrow range of our study population, which included only nonclinical, non-institutionalized, community-dwelling Japanese people aged 70 and 80 years. Although the sample was drawn from a complete enumeration of the resident record, most of the participants were physically and cognitively healthy. Indeed, it is possible that persons with dementia or those uninterested in health and/or its examination were spontaneously excluded from this study. Consequently, our results cannot be generalized to younger, older, or less healthy people. Caution is warranted in generalizing our findings to the rest of the Japanese population.

Our study is not a case-control study with patients, but a community based epidemiological study. Our study population included only non-institutionalized, community-dwelling, functionally independent people who were recruited from a complete enumeration of residents. Because retrospective examinations require good memory function, we excluded participants who were not cognitively able to complete memory tasks.

In our study, 3.0% of participants in the 70 years group and 3.9% in the 80 years group obtained scores that were lower than the mean scores minus 2 SDs in an age- and education-matched control group in another Japanese community-based study [38]. However, these percentages did not correspond with deterioration in cognitive function.

There are some newer masticatory performance assessment techniques that can be executed considerably fast, but may be highly resource-intensive for large populations. Nevertheless, it is important to consider that occlusal force is just another proxy indicator of the masticatory function, just like the masticatory performance, the masticatory efficiency, the perceived masticatory ability, among others; thus, a lot of information regarding the quality of the masticatory process was not being taken into account.

The MoCA-J measures comparative cognitive functional decline, including that associated with physiological aging. However, the conventional cut-off score for normal vs. abnormal (for example, dementia or MCI) has not yet been determined in the Japanese population [38]. However, the purpose of our study was to examine whether occlusal force was associated with global cognitive function, not with dementia or MCI, after adjusting for possible risk factors.

Another limitation is that our study was cross-sectional rather than longitudinal in nature. Thus, it is difficult to identify any causal relationships. It is impossible to capture individual functional decline using a cross-sectional method. In cross-sectional studies of dependent older people, it can be difficult to determine whether cognitive decline has led to the deterioration of oral health or if the opposite is true. Generally, persons with cognitive impairment may have a reduced ability to maintain oral hygiene, which could increase the risk of dental caries and periodontal disease, and consequently lead to tooth loss and limit the ability to chew hard food. Therefore, we attempted to limit the possible effect of day-to-day function on oral health by selecting only functionally independent old persons. The lack of significance in the relationship between cognitive function and the number of remaining teeth, after adjustment for confounding variables, does not support the direction of causality from reduced cognitive function to tooth loss. In addition, the participants reported their dietary habits for only the preceding month. Therefore, the history and changes in dietary habits over time, which can have powerful effects on the central nervous system, were unknown. Simple measurement did not reveal a clear association between periodontal status and CRP. Nevertheless, using path analysis, we attained a good fitting model with causal inferences about the relationships among various factors related to cognitive function. However, it is not possible to rule our reverse causation with a cross-sectional design. Further longitudinal study is needed to examine possible temporal relationships. Indeed, we are following up with the participants every 3 years to investigate the causal relationship between oral function and cognitive decline.

## Conclusion

After controlling for systemic inflammatory condition, depression status, dietary intake, and ApoE genotype in functionally independent elderly people, maximal occlusal force was positively associated with cognitive function directly as well as indirectly through dietary intake. These results suggest that decreased oral function might coincide with the early stages of cognitive decline.

## Supporting information

S1 Table Multivariable logistic regression models for occlusal force and MoCA-J score adjusted for significant independent variables. S1 Table.docx.
(DOCX)

S1 File Measurement of bilateral maximal occlusal force. S1 documents.docx.
(DOCX)

S2 File Minimal dataset. Minimal dataset.xslx.
(XLSX)
