# Supplementary material for: Occlusal force is correlated with cognitive function directly as well as indirectly via food intake in community-dwelling older Japanese: From the SONIC study
Source: PLoS One. 2018 Jan 5;13(1):e0190741. doi: 10.1371/journal.pone.0190741 (PMC5755890; doi:10.1371/journal.pone.0190741)
Supplement: S1 Table — docx. (DOCX) [file pone.0190741.s001.docx]

Appendix Table. Multivariable logistic regression models for occlusal force and MoCA-J score adjusted for significant independent variables.

| Independent variables | Model 3 | n=1623 | | |  |  | Model 4^§§^ | n=1323 | | |  |
| --- | --- | --- | --- | --- | --- | --- | --- | --- | --- | --- | --- |
|  | Adjusted  odds ratio | 95%CI | | | p-value |  | Adjusted  odds ratio | 95%CI | | | p-value |
| Age | .809 | .624 |  | 1.048 | .109 |  | .928 | .693 |  | 1.244 | .619 |
| Gender | .793 | .565 |  | 1.112 | .178 |  | .858 | .586 |  | 1.257 | .432 |
| Occlusal force | .896 | .856 |  | .939 | <0.001 |  | .912 | .867 |  | .959 | <0.001 |
| Education level |  |  |  |  | <0.001 |  |  |  |  |  | <0.001 |
| High school | .393 | .300 |  | .513 | <0.001 |  | 3.923 | 2.682 |  | 5.737 | <0.001 |
| College or more | .261 | .187 |  | .364 | <0.001 |  | 1.487 | 1.015 |  | 2.180 | .042 |
| Financial status |  |  |  |  | .002 |  |  |  |  |  | .003 |
| Fairly good | .650 | .485 |  | .873 | .004 |  | .640 | .462 |  | .886 | .007 |
| Good | .525 | .361 |  | .764 | .001 |  | .490 | .320 |  | .749 | .001 |
| Smoking history | .721 | .522 |  | .996 | .047 |  | .698 | .488 |  | 1.000 | .050 |
| Drinking habit | .947 | .704 |  | 1.273 | .717 |  | .911 | .654 |  | 1.270 | .582 |
| Hypertension | 1.279 | .962 |  | 1.700 | .090 |  | 1.235 | .897 |  | 1.701 | .195 |
| Diabetes | 1.182 | .868 |  | 1.609 | .290 |  | 1.197 | .815 |  | 1.760 | .359 |
| CRP>0.3 mg/dL | 1.458 | .999 |  | 2.128 | .051 |  | .580 | .381 |  | .883 | .011 |
| Depression | 1.185 | 1.063 |  | 1.321 | .002 |  | 1.212 | 1.071 |  | 1.372 | .002 |
| Green and yellow vegetables |  |  |  |  |  |  | .994 | .990 |  | .999 | .010 |
| Fruits |  |  |  |  |  |  | 1.000 | .997 |  | 1.003 | .991 |
| Meat |  |  |  |  |  |  | .999 | .990 |  | 1.007 | .739 |
| Grains |  |  |  |  |  |  | 1.000 | .998 |  | 1.002 | .949 |
| Nagelkerke R^2^ | 0.148 |  |  |  |  |  | 0.174 |  |  |  |  |

Model 3: Occlusal force (lowest quartile, upper 3 quartiles*) adjusted for gender (male*, female), education level (junior high school*, high school, college or more), self-rated financial status (poor*, fairly good, good), smoking history and drinking habits (yes, no*), hypertension (yes, no*), diabetes mellitus (yes, no*), overweight status (BMI: equal to or greater than, less* than 25 kg/m2), CRP (equal to or greater than, less* than 0.3 mg/dl) and Depression (GDS-5 score, continuous variable).

Model 4: Model 3 plus adjustments for food intake (continuous variable).

*: Reference category

§§: Participants currently receiving dietary counseling from a doctor or dietician, and those with intentional dietary change during the preceding year were excluded from analysis.
