# Supplementary material for: Occlusal force is correlated with cognitive function directly as well as indirectly via food intake in community-dwelling older Japanese: From the SONIC study
Source: PLoS One. 2018 Jan 5;13(1):e0190741. doi: 10.1371/journal.pone.0190741 (PMC5755890; doi:10.1371/journal.pone.0190741)
Supplement: S1 File — S1 documents.docx. (DOCX) [file pone.0190741.s002.docx]

Appendix

Measurement of bilateral maximal occlusal force

Bilateral maximal occlusal force was measured with pressure-sensitive sheets 98 microns thick (Dental Prescale, Type-R 50H, Fuji Film, Tokyo, Japan) that were analyzed using an image scanner (Occluzer, FPD-707, Fuji Film). Each pressure-sensitive sheet consisted of two polyethylene terephthalate films with numerous microcapsules with a colour-forming material between the films. Biting the sheet collapsed the microcapsules, and the colour agent contained in the capsules leaked out to chemically form a red colour. According to the strength of the pressure applied, different densities of colour were formed. The occlusal force was calculated after scanning the sheet with an image scanner (Occluzer), taking into consideration the occlusal contact area and different densities of color. The biting force (N) was determined as the sum of the degree of coloration and the area at each contact point. The occlusal force was measured as the sum of all occlusal contact sites in the intercuspal position including all regions (anterior and posterior regions) of the dental arch. This system measured the occlusal force per person and not that of the individual tooth or the dental arch.

The study participants performed maximal clenching in the intercuspal position with the pressure-sensitive film placed between the maxillary and mandibular dental arches. Participants with removable partial dentures kept their dentures in place during the measurement of the maximal occlusal force. To assess inter- and intra-examiner reliability in the study, each examiner measured occlusal force in five subjects and occlusal force five times in each subject for inter-examiner and intra-examiner reliability, respectively ([1](#_ENREF_1)).

As with MoCA-J scores, there is no authorized cut-off for normal vs. abnormal (dysfunction) occlusal force values. It simply serves as an index for comparative oral function.

1. Inomata C, Ikebe K, Kagawa R, Okubo H, Sasaki S, Okada T*, et al.* Significance of occlusal force for dietary fibre and vitamin intakes in independently living 70-year-old Japanese: from SONIC Study. J Dent. 2014;42:556-564.
